# Annotations

**Published:** 1892-08-27

**Authors:** 


### Annotations.

**Is Cholera Upon Us?**

News of an alarming character reaches us from Hamburg as we go to press. It was stated on Tuesday evening that cholera had broken out in all parts of the city. On Tuesday alone 340 fresh cases had occurred, and of that number no fewer than 130 had proved fatal, a mortality of more than one in three of those attacked. Several streets in the poorer part of the city have been closed against traffic. Dr. Koch and Dr. Rahts have been sent from Berlin by the Imperial Government, and Koch has pronounced several of the cases to be true Asiatic cholera. The presumption, therefore, is that an actual epidemic of that dreaded disease is now in progress at Hamburg, and Hamburg is practically at our own doors. It is true the German ocean lies between it and us; but there is daily communication by means of the large shipping traffic with London and other east of England ports. Worst of all is the fact that Hamburg is the principal distributing port for emigrants hastening from Russia and other Continental countries to our own shores and America. These are the bald facts as they reach us to-day (Wednesday). Everybody ought to be made aware of them, and everybody ought to be prepared to anticipate danger, and to deal with it, if it should come upon us, rationally and promptly. Nobody need die of mere fright. What we have all to do is to see to the scavenging of our houses, back yards, and streets at once; to burn all the refuse which can be burnt; to filter and boil all the water we drink, and rather to starve than to eat any kind of fruit, vegetables, meat, fish, or game which is tainted in the very least. If these things be done by private individuals, and the public authorities take all the precautions which they so well know how to take, and which they are quite prepared to put in hand, we may venture to turn our faces to the enemy with reasonable assurance. We have hot, "muggy" weather, and a malodorous river to contend with, otherwise we might probably dismiss all anxiety. In any case, to be forewarned is to be forearmed, and to avoid panic is to reduce the danger to a minimum.

**Hills or Sea?**

Now that almost everybody is off holiday-making, we may be quite sure that a considerable proportion of everybody is grumbling at the place in which they find themselves, and wishing they had gone elsewhere. This always happens, especially when people choose their holiday resort not from any characteristics of the place itself, but because the Browns were there last year and liked it so much. The Browns liked it, so the Smiths go; forgetting that the Browns are a sickly brood who could not endure the nipping and eager air for which the sturdy tribe of Smiths are pining, and therefore condemning themselves to a period of heat, midges, and *ennui*. "One man's meat is another man's poison," in air as well as in food; but to very few people does it occur, that so long as you go out of town, it matters little whether you go north or south, to the home of the east wind or the west, to the hills or to the sea. Yet, with a little thought, a little inquiry into one's constitution, it is easy enough to decide where to go. Mountain air is brisk, light, and energising; sea air is heavy and soporific. They are obviously adapted for different people. In that both are pure, they are beneficial to all city dwellers; but it does not follow that they are equally beneficial. For those who at home are confined to sedentary work, which does not, however, involve any special mental strain, the hills are best. If taste so prompts and funds permit, there is sport of some kind to give interest to the holiday, and lead the holiday maker to take exercise. It is wonderful how many miles a man will walk after grouse who, in London, takes a hansom from one end of Piccadilly to the other. It is wonderful how a man whose daily existence is that of a sybarite will lie on the heather for hours and creep in the misty dawn through bog and bracken, drawn by those antlers. that so often elude him after all. And even where the power or the desire to slay is lacking, there is that in a Briton's soul which prevents him from ever seeing a hill without determining to get to the top of it. Among the mountains we learn our strength, and unconsciously strengthen muscles that have dwindled for lack of use. But the power of the sea is different. Sea air is perhaps suited above all for those whose work is of the mind. It forces them to rest. You can't study hard by the sea—you are so apt to fall asleep in the middle, and on waking to find your thoughts determinedly fixing themselves on the next meal. You are reduced for the time to mere animalism, and that of the laziest and dullest type. Not a high ideal to aim at, this, it is true; but for the brain-worker, who lives on his nerve-energy, and drives himself into insomnia, it is, for at least a month in the year, the healthiest in the world.

**"Cures" for Habitual Drunkenness.**

Habitual drunkenness is now recognised as a disease, very properly, and the medical profession, aided as usual by an industrious army of benevolent quacks, is making strenuous efforts to deal with it by way of "cure." America appears determined to take the lead in this "forward movement," and the profession in that country has been very fruitful in remedies of both an open and a "secret" character. It will not surprise reasonable men that the so-called "specifics," chloride of gold among the number, have been, as a rule, complete failures. On the other hand, rational and common sense therapeutics, in the hands of men whose object is to cure their patients, and not to make of them experimental "corpora" for the purpose of spreading doctors' names abroad, are being everywhere increasingly successful. There are three things to be borne in mind in the treatment of inebriates: first, that alcoholism deranges the functions of most of the organs of the body at an early period, and therefore the initial thing to be done is to deal intelligently and thoroughly with the deranged functions. The second is to recognise that the inebriate is in many instances still a rational person, with a will and a conscience, and that his reason is to be appealed to and his will invoked for the saving of his own life and the well-being and comfort of his family. Many a drunkard, if rightly approached by the physician, and helped in a sympathetic spirit, will be his own best "physic bottle," and his own successful "specific." Thirdly, it is to be remembered that a large number of drunkards are naturally so weak in conscience and will, and have been reduced to so much lower a level than that at which Nature placed them, that neither drugs nor appeals to reason and conscience are of the least avail. With such as these there is only one rational method of procedure. They must be taken charge of, with their will or against their will; and they must be entirely and compulsorily deprived of all stimulants, and otherwise medically treated for a sufficient length of time for the habit and craving, which are their worst enemies, to be entirely broken. These are the methods of reason and honesty. The *British Medical Journal* tells us that they are still by far the most successful of all methods. We are weary of faddists and self-seekers on this subject. If the Colleges of Physicians would bestir themselves in the way of issuing authoritative directions, and the legislatures would give to responsible relatives and friends adequate powers of compulsory detention, habitual drunkenness would soon become a thing of the past.

